# Microsporidia: Why Make Nucleotides if You Can Steal Them?

**DOI:** 10.1371/journal.ppat.1005870

**Published:** 2016-11-17

**Authors:** Paul Dean, Robert P. Hirt, T. Martin Embley

**Affiliations:** Institute for Cell and Molecular Biosciences, The Medical School, Newcastle University, Newcastle-upon-Tyne, United Kingdom; Boston College, UNITED STATES

## Abstract

Microsporidia are strict obligate intracellular parasites that infect a wide range of eukaryotes including humans and economically important fish and insects. Surviving and flourishing inside another eukaryotic cell is a very specialised lifestyle that requires evolutionary innovation. Genome sequence analyses show that microsporidia have lost most of the genes needed for making primary metabolites, such as amino acids and nucleotides, and also that they have only a limited capacity for making adenosine triphosphate (ATP). Since microsporidia cannot grow and replicate without the enormous amounts of energy and nucleotide building blocks needed for protein, DNA, and RNA biosynthesis, they must have evolved ways of stealing these substrates from the infected host cell. Providing they can do this, genome analyses suggest that microsporidia have the enzyme repertoire needed to use and regenerate the imported nucleotides efficiently. Recent functional studies suggest that a critical innovation for adapting to intracellular life was the acquisition by lateral gene transfer of nucleotide transport (NTT) proteins that are now present in multiple copies in all microsporidian genomes. These proteins are expressed on the parasite surface and allow microsporidia to steal ATP and other purine nucleotides for energy and biosynthesis from their host. However, it remains unclear how other essential metabolites, such as pyrimidine nucleotides, are acquired. Transcriptomic and experimental studies suggest that microsporidia might manipulate host cell metabolism and cell biological processes to promote nucleotide synthesis and to maximise the potential for ATP and nucleotide import. In this review, we summarise recent genomic and functional data relating to how microsporidia exploit their hosts for energy and building blocks needed for growth and nucleic acid metabolism and we identify some remaining outstanding questions.

## Introduction

Microsporidia are fungi-related eukaryotic parasites with over 1,400 reported species that infect a wide range of hosts including humans, mammals, and insects [1,2]. They are all strict obligate intracellular parasites and can only complete their life cycle within an infected eukaryotic host cell. The life cycle (S1 Fig) of a typical microsporidia begins with the germination of a resistant spore that physically injects the sporoplasm into the host cell through a polar tube [1]. The parasite cell (meront) grows and multiplies within the host cell cytoplasm through several rounds of division and differentiates into a spore, which exits the host cell, typically through host cell lysis, to complete the cycle (S1 Fig).

**Fig 1:**
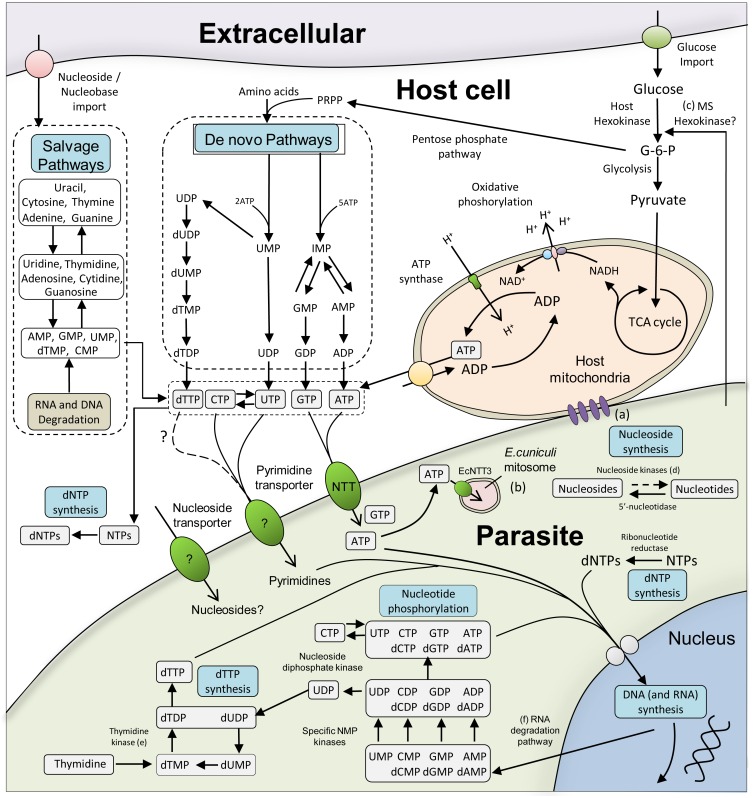
Nucleotide acquisition and metabolism in host cells and microsporidia. Schematic illustration showing nucleotide metabolism in a typical microsporidian parasite within an infected host cell. Host cells can make nucleotides via *de novo* biosynthesis and regenerate ATP by oxidative phosphorylation—these pathways are absent in microsporidia [5]. Host purine nucleotides can be stolen using microsporidia NTT transporters and then efficiently used and recycled by the parasites [18]. Key: (a) *E*. *cuniculi* physically tethers mitochondria using an unidentified protein [49]. (b) Only EcNTT3 of *E*. *cuniculi* has been found in the mitosome [38]. (c) *Nematocida* may secrete a hexokinase into the host cell to stimulate host nucleotide production [6]. (d) Nucleoside kinases are apparently absent from some microsporidian genomes but are present in *Trachipleistophora hominis* [18]. (e) Thymidine kinase is present in some microsporidia but not all [18]. (f) The microsporidian RNA degradation pathway is shown in S2 Fig.

Microsporidian genomes have some of the smallest coding capacities among eukaryotes [3] and analyses reveal that they have lost many of the biosynthetic genes needed for making basic metabolites, such as the nucleotides required for making DNA and RNA, amino acids for making proteins, and lipids for making membranes [4]. Genome analyses also show that all microsporidia have lost the pathways for oxidative phosphorylation and the tricarboxylic acid (TCA) cycle [5,6] and *Enterocytozoon bieneusi*, a major pathogen of immunocompromised patients, has also lost glycolysis [7]. So the capacity for independent biosynthesis of ATP appears to be very limited in microsporidia. Since a typical cell requires enormous amounts of ATP (10^7^ ATP molecules per second) to grow and divide [8], actively growing microsporidia must impose a very high demand for ATP on infected host cells. In this review, we summarise what is currently known about the transport proteins and mechanisms (summarised in Fig 1) that are used to acquire the ATP and other nucleotides needed to support the intracellular growth and replication of this enormously successful group of eukaryotic parasites.

### Nucleotide metabolism and availability within the host cell

Nucleotides are the building blocks of DNA and RNA that are essential to all life. In free-living species, the eight major purine or pyrimidine nucleoside triphosphates (Box 1) can either be synthesised *de novo* from amino acids or recycled (salvaged) from the rapid turnover of RNA using pathways that are conserved among prokaryotes and eukaryotes [9,10]. While nucleotide synthesis is located in the cytoplasm, nucleotides can freely diffuse into the eukaryotic nucleus [11], possibly explaining how some microsporidia can complete their lifecycle in the host nucleus [12,13].

**Box 1. Facts and Figures: Nucleotides, nucleosides, and their cellular concentrations and bacterial and microsporidian NTTs**

**ATP demand in cells:** It is estimated that 50 ATP are used to make one nucleotide from scratch when the ATP needed to make all of the required co-factors and substrates is also included [7,14]. Around 10^7^ ATP per second is used in a typical cell [7,14]

**Nucleotides:** There are four major types of **nucleotide triphosphates** that make up RNA (ATP, GTP, CTP, UTP) and four that make DNA (dATP, dGTP, dCTP, dTTP). When DNA and RNA are degraded, the nucleoside monophosphate is released which can be recycled.

Nucleotides are made of 3 components:

- Purine (adenine, guanine) or pyrimidine (cytosine, uracil or thymine) base
- A ribose or deoxyribose sugar
- One to three phosphate groups

**Nucleosides (adenosine, guanosine, cytidine, uridine, thymidine**): Similar to nucleotides but do not have the phosphate groups.

**Intracellular concentrations:** Mean nucleotide concentrations in a mammalian cell [10]: ATP (3000 μM), GTP (500 μM), UTP (600 μM), CTP (300 μM).

Deoxyribonucleotide (dNTP) and nucleoside concentrations: 4–40 μM.

**Nucleotide transporter (NTTs) affinities:** Apparent K_m_ (ATP) of NTTs from the microsporidian *E*. *cuniculi* NTTs [37] are: EcNTT1 = 11.4 μM, EcNTT1 = 19.8 μM, EcNTT1 = 24.2 μM, EcNTT1 = 2.6 μM. Apparent K_m_ (ATP) of bacterial NTTs are: Protochlamydia amoeabophila *PamNTT1 = 95 μM, PamNTT2 = 437 μM, PamNTT5 = 360 μM; *Chlamydia trachomatis*: Npt1_Ct_ = 48 μM, Npt2_Ct_ = 1158 μM; RpTLC = 100 μM [40–43].

* Note, the apparent K_m_ (ATP) for PamNTT1 in liposomes was 17–100 μM, depending on intra-liposomal nucleotide concentration [43]).

Nucleotide concentrations in mammalian cells have been well documented [11], with ribonucleoside triphosphates (NTPs), particularly ATP (Box 1) [11], at highest concentrations. The concentrations of di- and monophosphate forms of nucleotides, of nucleosides, and of nucleobases are all less than <5μM compared to ~3000 μM for ATP [11] (Box 1). The deoxyribonucleotides (dNTP) that are the building blocks of DNA are also at considerably lower concentrations in a cell than the corresponding NTPs, possibly explaining why microsporidia have retained the ability to synthesise their own dNTPs [4] providing they have a source of NTPs from the host cell. Tight control over dNTP synthesis is also critical to cell viability [14] as discussed below.

### Nucleotide biosynthesis pathways and the microsporidia

**Fig 2:**
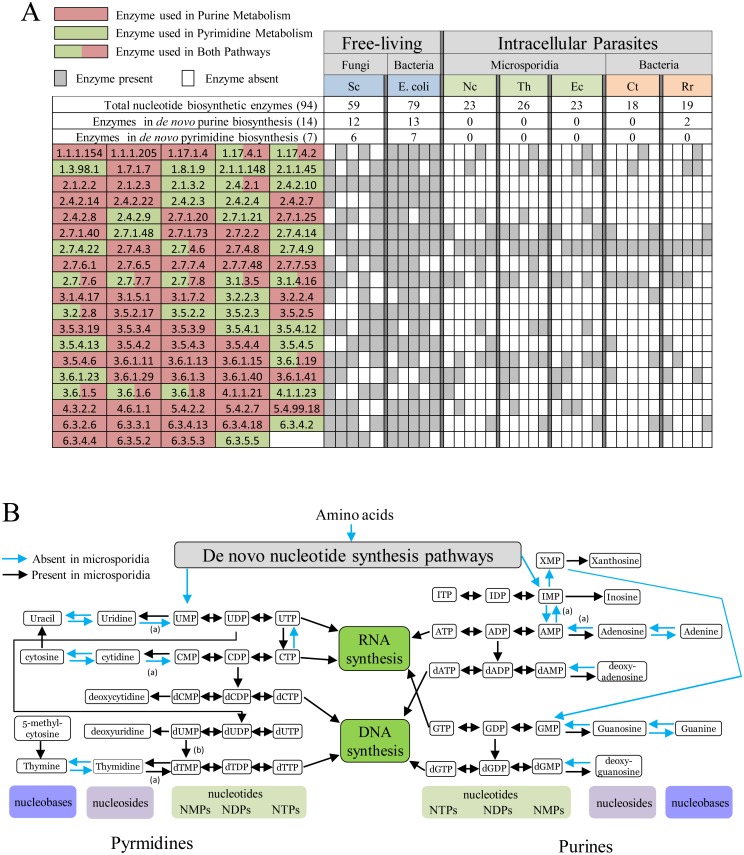
Nucleotide biosynthesis in microsporidia. (A) Nucleotide biosynthetic enzymes found in the genomes of yeast, *Escherichia coli*, Microsporidia, and intracellular pathogenic bacteria for purine (red) and pyrimidine (green) metabolism according to the KEGG database [60] are shown along with respective EC numbers (see S3 Fig). Sc = *Saccharomyces cerevisiae*; Th = *Trachipleistophora hominis*; Ec = *Encephalitozoon cuniculi*; Ct = *Chlamydia trachomatis*; Rr = *Rickettsia rickettsii*. (B) Purine and pyrimidine pathways retained in microsporidia genomes (black arrows) enable recycling of all of the major nucleotides based on available genome data [18]. Some exceptions to the general rule can be found in (a) *T*. *hominis* or (b) *N*. *ceranae*, as described in the text.

**Fig 3:**
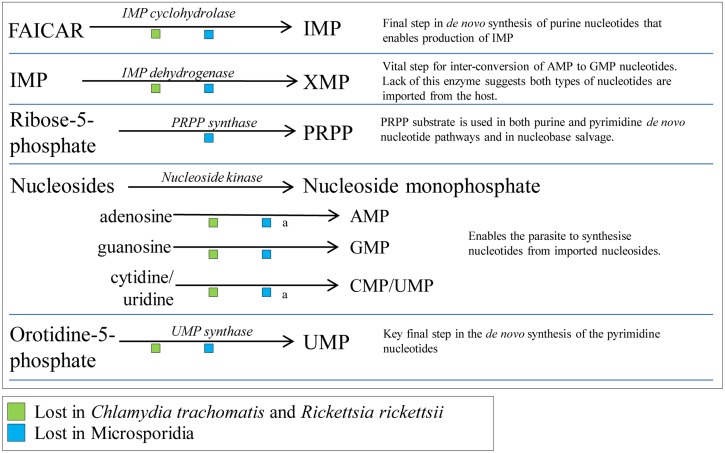
Key metabolic steps lost by microsporidia and intracellular bacteria. Several key enzymes and the associated pathways are shown that have been lost during microsporidian evolution (green square). The implications for the parasites are shown to the right. With the exception of PRPP synthase, all enzymes have been lost by obligate intracellular bacterial pathogens (green square). Exceptions: (a) nucleoside kinases are retained in *T*. *hominis*

Making nucleotides *de novo* from amino acids is energy expensive and is estimated at around 50 ATP per nucleotide when the costs of co-factors and substrates are included [15]. Microsporidian genomes lack the enzymes needed for the *de novo* synthesis of nucleotides [4,6] (Figs 2 and 3) including phosphoribosyl pyrophosphate (PRPP) synthase [4], which makes the substrate PRPP that is required for the activation of ribose-5-phosphate for both purine and pyrimidine *de novo* pathways [16,17]. The loss of this biosynthetic function alone means *de novo* synthesis is not possible. Other key enzymes are also absent [18] (Fig 3), including IMP cyclohydrolase that makes inosine monophosphate (IMP)—the first purine nucleotide in the *de novo* pathway—and UMP synthase [18], which makes the first pyrimidine nucleotide, uridine monophosphate (UMP). These enzymes are also missing in obligate intracellular bacteria [18] (Figs 2 and 3), indicating that *de novo* nucleotide synthesis is also not possible in these organisms. However, upon obtaining nucleotides from the host, microsporidia and these bacterial pathogens have retained the core suite of enzymes for metabolising and recycling nucleotides (Fig 2B) [18].

#### Inter-converting purine nucleotides

In free-living species, the first purine nucleotide produced by *de novo* synthesis is IMP, which requires hydrolysis of six ATP, and can then be converted to either AMP or guanosine monophosphate (GMP), which requires hydrolysis of an additional ATP. Genome analyses [4,6,18] suggest that microsporidia lack the enzymes for inter-converting IMP to AMP or GMP, or for inter-converting between guanosine and adenosine nucleotides [4,6,18], suggesting that they must import both types of purine nucleotide. In addition, the apparent lack of a GMP synthase or adenylosuccinate synthase in the genome sequences of microsporidia [4] also suggests that import of inosine nucleotides (IMP/IDP/ITP) would be fruitless as they cannot be utilised. Microsporidia have, however, retained all of the necessary enzymes for converting between the three phosphorylation states of guanosine and adenosine nucleotides, including the broad-spectrum nucleoside diphosphate kinase (NDK/YNK in yeast), which is highly expressed during infection and in the spore stage of the microsporidian *Trachipleistophora hominis* [19,20], suggesting an important role in parasite metabolism.

#### Inter-converting pyrimidine nucleotides

The first pyrimidine nucleotide produced by the *de novo* pathway in free-living species is UMP, which can be stepwise converted into the pyrimidine triphosphates dTTP, UTP, or CTP (Fig 1). Analysis of microsporidian genomes suggests that some species are unable to convert CTP and its derivatives to UTP as they lack the relevant deaminase enzymes [18], suggesting that they need to import UTP from the host. Some microsporidians have retained the enzyme CTP synthase enabling them to make CTP from imported UTP as a route towards synthesising cytidine derivatives [4,6]. However, this step is energy expensive and, therefore, an ability to steal host CTP would be beneficial; hence, some intracellular *Chlamydia* possess a CTP synthase and can also import CTP from the host cell [21]. Microsporidia have generally retained all of the enzymes [18] needed to make dTTP from UTP, as described below. In summary, if UTP can be stolen from the host, microsporidia appear to have the necessary enzymes to synthesise the other pyrimidines.

#### Synthesis of dNTPs

Maintaining the correct cellular concentrations of the deoxyribonucleotides (dNTPs) that are needed to make DNA is critical as an imbalance can be highly mutagenic [14,22–24]. Control over cellular dNTP concentrations is exerted at the level of the broad-spectrum enzyme ribonucleotide reductase, which is tightly regulated by the dNTPs themselves [12]. The need to exert tight control over dNTP concentrations may explain why microsporidia [4,18], along with the obligate intracellular bacteria *Chlamydia* [25] and *Rickettsia* [18], have retained their own ribonucleotide reductase and, hence, do not depend on the relatively low levels of dNTPs in the host cytoplasm [11].

Unlike the other dNTPs, dTTP must be synthesised from dUMP and requires the enzyme thymidylate synthase that is found in some, but not all, microsporidia [18]. The apparent absence of thymidylate synthase in *Nosema ceranae* (and potentially other microsporidians that lack this enzyme), suggests they must make or acquire dTTP by other means. Indeed, several species of microsporidia, including *N*. *ceranae* and *Encephalitozoon cuniculi* [26], have acquired the enzyme thymidine kinase by lateral gene transfer from bacteria and this would allow them to phosphorylate thymidine imported from the host cell [26].

### Microsporidia and the recycling of nucleotides

The continual turnover of RNA, which releases the nucleoside monophosphates, represents a major, ready-made source of nucleotides [27]. The enzymes needed to regenerate nucleoside triphosphates following nucleic acid degradation have been retained by microsporidia [4] and include dedicated monophosphate kinases and the broad-spectrum nucleoside diphosphate kinase (NDK/YNK in yeast) (Fig 1) [4,18]. While nucleoside triphosphate regeneration involves some energy-expensive steps, it still represents a more economical source of nucleotides compared to *de novo* nucleotide synthesis.

In eukaryotic cells, RNA degradation occurs by two main pathways [28] (S2 Fig), both of which are initiated by the deadenylation of RNA by enzymes including the Ccr4 complex. The machinery for both RNA degradation pathways is present in microsporidia (S2 Fig) including the Ccr4 complex [4]; the decapping enzymes Dcp1 and Dcp2 [29]; the exonuclease Rat1, which is involved in 5’ to 3’ degradation; and the exosomal complex needed for 3’ to 5’ degradation, including the exosomal protein Dis3, which acts as the main catalytic component of the exosome complex in yeast [27,30].

In comparison to other organisms, there are some important salvage enzymes missing from microsporidian genomes (Fig 2) [31]. For example, microsporidia appear to lack [18] the various enzymes for conversion of nucleobases to nucleotides, suggesting that they cannot use nucleobases as starting points for nucleotide synthesis. In addition, the ribose-phosphate moiety of nucleotides that represents a potential carbon or energy source [31] is unlikely to be recycled in microsporidia as it must be converted to intermediates for glycolysis [16]—a pathway that appears to be most active in the spore stage of microsporidia [19,32] or has been lost altogether [7].

Genome analysis suggests that microsporidia have retained the broad-spectrum enzyme 5'-nucleotidase that converts nucleotides to nucleosides, a known regulatory function of this enzyme, thus maintaining an optimal nucleotide balance required for normal cell physiology [24,33]. By contrast, some microsporidia are unable to convert nucleosides to nucleotides [18] as they lack the necessary kinases, and this raises doubt about whether these species could utilise nucleosides imported from the host. By contrast, *T*. *hominis* has retained several nucleoside kinases [18] that would enable it to utilise nucleosides stolen from the host or generated internally by the activity of the 5'-nucleotidase.

### How do microsporidia acquire the energy and nucleotides they need?

Nucleotides cannot be transported physiologically across plasma membranes without specific transporters [34, 35]. In mammalian cells, extracellular nucleotides are generally converted to nucleosides that can then be imported by members of the equilibrative nucleoside transporter (ENT) family [34]. Members of the ENT family are found in some parasites, but, so far, are not found in microsporidia [4,35]. Instead, microsporidia use a family of nucleotide transporters (NTTs) that are also found in phylogenetically diverse intracellular bacterial pathogens, such as *Chlamydia* and *Rickettsia* [36], to import nucleotides directly from the host cell cytoplasm.

Phylogenetic analyses [18,37,38] suggest that a single NTT gene was probably acquired by horizontal transfer from bacteria into the microsporidian common ancestor. This was followed by lineage-specific gene duplications to generate the multiple copies of NTT genes found in contemporary microsporidian genomes [4,6,18]. For example, the microsporidia *T*. *hominis*, *E*. *cuniculi*, and *E*. *bieneusi* have four NTTs while *Spraguea lophii* has six [39]. NTT gene duplications may provide the parasites with the starting materials for NTT functional diversification, differential NTT expression throughout the life cycle, or a gene dosage effect to increase the amount of NTTs being made.

Microsporidian NTTs have been found to be highly and differentially expressed during the different stages of the microsporidia life cycle [6,20] including spores [19]. In *E*. *cuniculi*, one of its four NTTs is localised to its highly reduced mitochondrion (called a mitosome [40]), whereas the other three NTTs are located at the parasite cell surface [38]. By contrast, all four NTTs of *T*. *hominis* are located at the cell surface [18], suggesting that the location of NTT transporters at the host–parasite interface is a general strategy used by microsporidia to exploit host cells [18,38].

The lack of axenic culture systems and the strict obligate intracellular lifestyle of microsporidia has impeded attempts to genetically manipulate these parasites [41], and all of the published functional work with microsporidian NTTs has employed heterologous expression in engineered *E*. *coli* strains (Box 1) [18,38]. This work has shown that the four NTTs in *E*. *cuniculi* and *T*. *hominis* can all transport ATP [18,38] and, hence, they can, in principal, be used to steal vital energy from the infected host cell. The *T*. *hominis* NTTs can also transport other purine nucleotides (ADP, GTP, and guanosine diphosphate [GDP]) that are needed for DNA and RNA biosynthesis, but not pyrimidine nucleotides [18]. Dose response data suggests that the four *E*. *cuniculi* NTTs [38] have a high affinity for ATP, with apparent K_m_ values considerably lower than that of bacterial NTTs [42–44] (Box 1), and well below host cytosolic ATP concentrations [11]—implying a high level of ATP transport could occur during infection.

The lack of transport of pyrimidine nucleotides by *T*. *hominis* NTTs expressed in *E*. *coli* raises the question of how *T*. *hominis* obtains the pyrimidines needed to make DNA and RNA. As discussed above, it appears that all major pyrimidine nucleotides can generally be synthesised if UTP is available, but no UTP transport was detected by the *T*. *hominis* NTTs [18]. It is not yet clear if an inability to transport pyrimidine nucleotides is a general feature of microsporidian NTTs because transport of radiolabelled pyrimidine nucleotides has only been tested for *T*. *hominis* [18,38]. Substrate competition experiments with the *E*. *cuniculi* NTTs suggest that ATP transport is not reduced by cold competitor pyrimidine nucleotides and, hence, pyrimidine transport appears unlikely [38]. However, since some bacterial NTTs can transport both purine and pyrimidine nucleotides [45] as well as NAD [46], it would not be too surprising if microsporidian NTTs in different species have also evolved to transport different substrates, including pyrimidine nucleotides. The extraordinary possibilities of NTT-mediated transport were recently demonstrated when a diatom NTT was used to import unnatural nucleotides into an engineered *E*. *coli* strain to create a semisynthetic organism with an expanded genetic alphabet [47].

It has been suggested [6] that homologues of the bacterial-like NupG transporters that are conserved on all microsporidian genomes [4] might, as in *E*. *coli*, transport purine and pyrimidine nucleosides including adenosine and uridine [48]. However, there is currently no functional data for microsporidia NupG-like transporters and genome analysis suggest that some microsporidia would also be unable to convert imported nucleosides into nucleotides as they lack the necessary nucleoside kinases [18] (Figs 2 and 3). In common with other parasites, microsporidia possess genes for a number of transporter families and hypothetical transporters [4,19,35] whose locations and functions are currently unknown. Given the minimal nature of microsporidian primary metabolism, it would not be surprising if some of these putative transport proteins also played roles in providing the metabolites needed for parasite growth, DNA biosynthesis, and RNA biosynthesis.

### Manipulating the host cell to support nucleotide acquisition

While NTTs confer an ability to steal nucleotides, the absolute dependence on host nucleotides could potentially act as an Achilles’ heel for microsporidia if the host was able to limit their availability. Recent work now suggests that microsporidia have evolved strategies to manipulate host cells and to ensure that a ready supply of host nucleotides is maintained.

Stimulating nucleotide metabolism in the host is one obvious strategy to increase the available pools of energy and nucleotides for import and there is some preliminary data that hints at how microsporidia could possibly do this. RNAseq analysis of the microsporidian *Nematocida parisii* during infection of the nematode *Caenorhabditis elegans* demonstrated the upregulation of a microsporidian hexokinase during early infection, despite low expression of other glycolytic enzymes, suggesting it may have an alternative role [6]. The presence of a signal peptide in this hexokinase, which was not found in the other glycolytic enzymes in this species [6], raised the possibility that it might be secreted into the host. The presence of a functional secretion signal was supported when the protein was expressed in yeast [6]. Hexokinase catalyses the phosphorylation of glucose to glucose-6-phosphate that can be used to synthesise PRPP and other nucleotide biosynthetic precursors. Thus, secretion of a microsporidian hexokinase could, in principal, stimulate host nucleotide production [6]

Mitochondria are rich sources of ATP and the microsporidian *E*. *cuniculi* forms an intimate association with host mitochondria [49,50], possibly to maximise the surface area in contact between parasite and organelle. Mitochondria appear to be physically attached to the *E*. *cuniculi* parasitophorous vacuole [49] suggesting that it may be porous to ATP or has associated transport proteins to permit ATP passage [51]. Association with host mitochondria has also been reported for intracellular bacterial parasites including *Legionella* [52] and *Chlamydia* [53], as well as the microbial eukaryote *Toxoplasma* [54], which secretes a protein called MAF1 to tether mitochondria to the parasite surface [54]. Homologues of MAF1 are not present in the *E*. *cuniculi* genome [49], but electron microscopy images suggest that *E*. *cuniculi* does use electron-dense proteinaceous structures to tether mitochondria and that it also influences the location of mitochondrial ATP-gating channels in the outer mitochondrial membrane [49]. Whether microsporidia can also increase ATP production by host mitochondria is not known, but no changes in mitochondrial activity were detected during infection by *E*. *cuniculi* [49,50]. Interestingly, however, comparing the transcriptome of *T*. *hominis*-infected mammalian host cells with non-infected controls did suggest that host-energy metabolism and mitochondrial biogenesis were both induced upon infection [20].

### Future perspectives

Comparative genomics data suggests that microsporidians cannot make their own nucleotides and must, therefore, import them from infected host cells. Given the central and non-redundant role of nucleotide import and metabolism in the parasite life cycle, this makes it a logical therapeutic target, especially against enzymes and transporters that are not found in host species. These would include the bacterial-derived NTT nucleotide transporters that appear to be so critical for microsporidia growth and replication, which are not found in vertebrates. Since NTT transporters are also used by important bacterial pathogens like *Chlamydia* and *Rickettsia* to exploit the eukaryotic host cells that they infect, finding effective therapeutic agents that act against NTTs would have applied interest beyond microsporidia, although the presence of multiple copies of such transporters in Microsporidia and their overlapping substrate ranges may complicate or hinder such approaches. Functional work on the other transporters present in microsporidian genomes to establish which are surface-located and which substrates they transport will also be important for filling the many gaps in our understanding of microsporidia–host interdependencies, including the acquisition of pyrimidine nucleotides needed for parasite DNA and RNA biosynthesis. Experimental work is essential to identify substrate specificities given the highly derived nature of microsporidia DNA and protein sequences and the resultant difficulty in reliably predicting protein function based upon low sequence similarity to characterised proteins from model organisms.

The mechanism of transport used by microsporidian NTTs has not been determined. NTT transporters in bacteria can function as ATP/ADP exchangers [42,45] to provide energy, or as proton-driven symporters to provide net import of nucleotides for DNA and RNA synthesis [42,45]. It is currently unclear if microsporidian NTTs also use different transport mechanisms, but given the demands imposed by parasite growth and replication, we think it very likely that both symporters and exchangers have also evolved during microsporidia NTT evolution. Understanding how NTTs function in detail would obviously be aided by the availability of a high-resolution structure for one or more NTT proteins, but unfortunately none are yet available. In particular, the levels of expression of microsporidian NTT proteins in *E*. *coli* are very low so providing enough protein for structural and comprehensive liposome studies [45] will require systematic investigation of different strategies to improve protein yields and the evaluation of eukaryotic [55], as well as prokaryotic expression systems.

Bacterial intracellular pathogens are known to utilise diverse effector proteins to manipulate the metabolism of the host cells that they infect [56,57]. It is very likely that microsporidia also use a variety of strategies and secreted proteins to manipulate host cellular processes, including energy and nucleotide metabolism. At present, it is difficult to investigate these phenomena effectively because of the lack of tools for reproducible genetic manipulation of well described microsporidian model species. However, some progress has recently been made using RNAi on the honeybee microsporidian parasite *N*. *ceranae* [58,59]. If the promise of these initial experiments can be confirmed and extended to more tractable model species, it might finally be possible to test hypotheses of microsporidian protein function and their potential role(s) in microsporidia–host interactions at the molecular level.

## Supporting Information

S1 Fig The lifecycle of a microsporidian. A typical life cycle begins with the germination of a spore, which discharges a polar tube that pierces the host cell plasma membrane enabling transfer of the parasite sporoplasm into the host cytoplasm. The parasite cell (meront) grows and divides, sometimes within a parasitophorous vacuole (not shown), and after several rounds of division, differentiates back into spores, which are released following host cell lysis. The life cycle of T. hominis during infection of cultured cells, is around 3–4 days.
(PDF)

S2 Fig RNA degradation pathways in microsporidia enable recycling of nucleotides. RNA (mRNA and rRNA) is a rich source of nucleotides that can be continually recycled in the cell. Genome analysis [4] suggest microsporidia have retained components needed for RNA degradation via 2 main pathways (a) 3’>5’ degradation involving the exosomal complex (b) 5’>3’ degradation involving decapping enzymes and Rat1. All enzymes depicted here are conserved in at least 9 microsporidian genomes [4].
(PDF)

S3 Fig Enzymes involved in purine and pyrimidine nucleotide biosynthesis and their EC numbers as given in Fig 2A in the main text. (PDF)
