# Supplementary figures and images for: Microsporidia: Why Make Nucleotides if You Can Steal Them?
Source: PLoS Pathog. 2016 Nov 17;12(11):e1005870. doi: 10.1371/journal.ppat.1005870 (PMC5113988; doi:10.1371/journal.ppat.1005870)

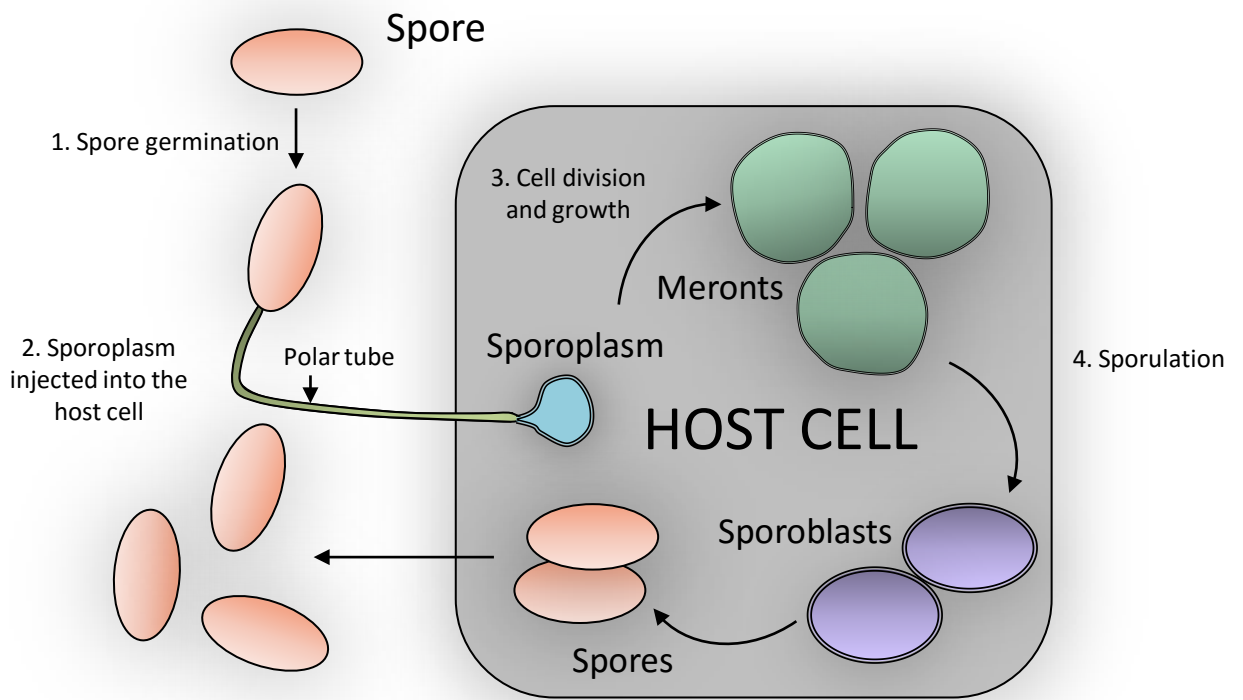

Supplement: S1 Fig — A typical life cycle begins with the germination of a spore, which discharges a polar tube that pierces the host cell plasma membrane enabling transfer of the parasite sporoplasm into the host cytoplasm. The parasite cell (meront) grows and divides, sometimes within a parasitophorous vacuole (not shown), and after several rounds of division, differentiates back into spores, which are released following host cell lysis. The life cycle of T. hominis during infection of cultured cells, is around 3–4 days. (PDF) [file ppat.1005870.s001.pdf]

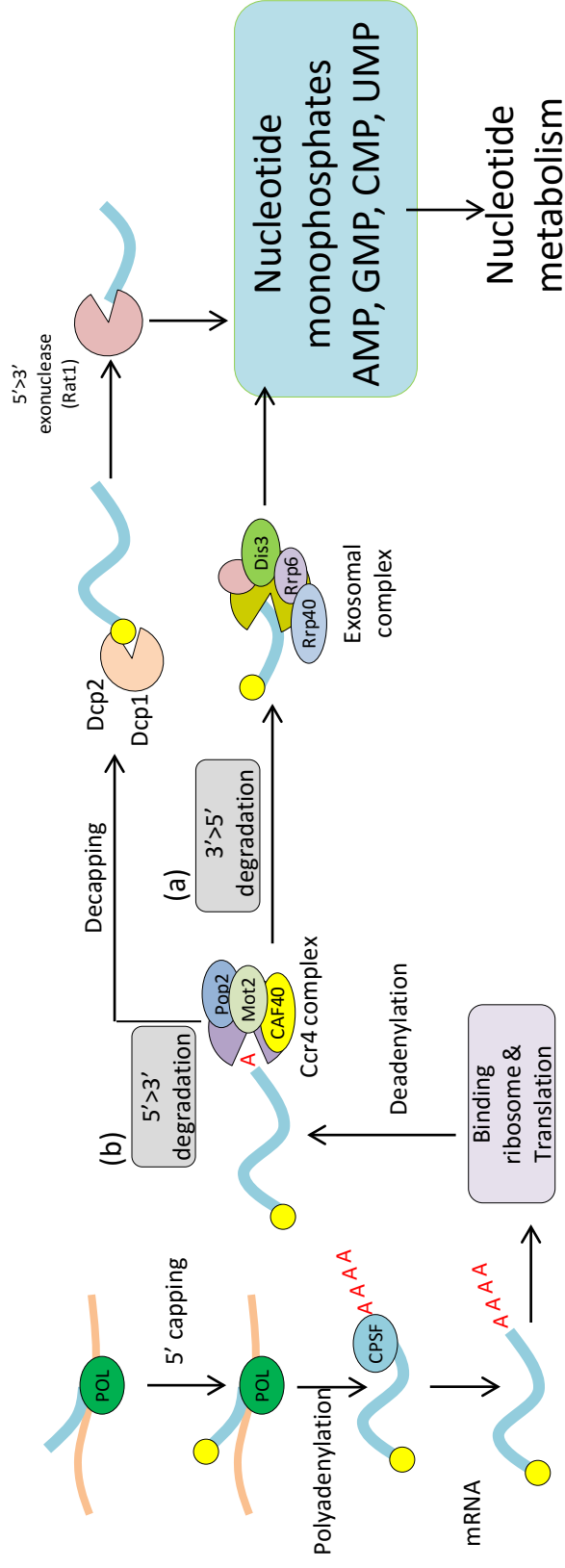

Supplement: S2 Fig — RNA (mRNA and rRNA) is a rich source of nucleotides that can be continually recycled in the cell. Genome analysis [4] suggest microsporidia have retained components needed for RNA degradation via 2 main pathways (a) 3’>5’ degradation involving the exosomal complex (b) 5’>3’ degradation involving decapping enzymes and Rat1. All enzymes depicted here are conserved in at least 9 microsporidian genomes [4]. (PDF) [file ppat.1005870.s002.pdf]
