# Supplementary material for: Microsporidia: Why Make Nucleotides if You Can Steal Them?
Source: PLoS Pathog. 2016 Nov 17;12(11):e1005870. doi: 10.1371/journal.ppat.1005870 (PMC5113988; doi:10.1371/journal.ppat.1005870)
Supplement: S3 Fig — (PDF) [file ppat.1005870.s003.pdf]

1.1.1.154	Ureidoglycolate dehydrogenase.
1.1.1.205	IMP dehydrogenase.
1.17.1.4	Xanthine dehydrogenase.
1.17.4.1	Ribonucleoside-diphosphate reductase.
1.17.4.2	Ribonucleoside-triphosphate reductase.
1.3.98.1	Dihydroorotate oxidase (fumarate).
1.7.1.7	GMP reductase.
1.8.1.9	Thioredoxin-disulfide reductase.
2.1.1.148	Thymidylate synthase (FAD).
2.1.1.45	Thymidylate synthase.
2.1.2.2	Phosphoribosylglycinamide formyltransferase.
2.1.2.3	Phosphoribosylaminoimidazolecarboxamide formyltransferase.
2.1.3.2	Aspartate carbamoyltransferase.
2.4.2.1	Purine-nucleoside phosphorylase.
2.4.2.10	Orotate phosphoribosyltransferase.
2.4.2.14	Amidophosphoribosyltransferase.
2.4.2.22	Xanthine phosphoribosyltransferase.
2.4.2.3	Uridine phosphorylase.
2.4.2.4	Thymidine phosphorylase.
2.4.2.7	Adenine phosphoribosyltransferase.
2.4.2.8	Hypoxanthine phosphoribosyltransferase.
2.4.2.9	Uracil phosphoribosyltransferase.
2.7.1.20	Adenosine kinase.
2.7.1.21	Thymidine kinase.
2.7.1.25	Adenylyl-sulfate kinase.
2.7.1.40	Pyruvate kinase.
2.7.1.48	Uridine kinase.
2.7.1.73	Inosine kinase.
2.7.2.2	Carbamate kinase.
2.7.4.14	UMP/CMP kinase.
2.7.4.22	UMP kinase.
2.7.4.3	Adenylate kinase.
2.7.4.6	Nucleoside-diphosphate kinase.
2.7.4.8	Guanylate kinase.
2.7.4.9	dTMP kinase.
2.7.6.1	Ribose-phosphate diphosphokinase.
2.7.6.5	GTP diphosphokinase.
2.7.7.4	Sulfate adenylyltransferase.
2.7.7.48	RNA-directed RNA polymerase.
2.7.7.53	ATP adenylyltransferase.
2.7.7.6	DNA-directed RNA polymerase.
2.7.7.7	DNA-directed DNA polymerase.
2.7.7.8	Polyribonucleotide nucleotidyltransferase.
3.1.3.5	5'-nucleotidase.
3.1.4.16	2',3'-cyclic-nucleotide 2'-phosphodiesterase.
3.1.4.17	3',5'-cyclic-nucleotide phosphodiesterase.
3.1.5.1	dGTPase.
3.1.7.2	Guanosine-3',5'-bis(diphosphate) 3'-diphosphatase.
3.2.2.3	Uridine nucleosidase.
3.2.2.4	AMP nucleosidase.
3.2.2.8	Ribosylpyrimidine nucleosidase.
3.5.2.17	Hydroxyisourate hydrolase.
3.5.2.2	Dihydropyrimidinase.
3.5.2.3	Dihydroorotase.
3.5.2.5	Allantoinase.
3.5.3.19	Transferred entry: 3.5.1.116.
3.5.3.4	Allantoicase.
3.5.3.9	Allantoate deiminase.
3.5.4.1	Cytosine deaminase.
3.5.4.12	dCMP deaminase.
3.5.4.13	dCTP deaminase.
3.5.4.2	Adenine deaminase.
3.5.4.3	Guanine deaminase.
3.5.4.4	Adenosine deaminase.
3.5.4.5	Cytidine deaminase.
3.5.4.6	AMP deaminase.
3.6.1.11	Exopolyphosphatase.
3.6.1.13	ADP-ribose diphosphatase.
3.6.1.15	Nucleoside-triphosphate phosphatase.
3.6.1.19	Nucleoside-triphosphate diphosphatase.
3.6.1.23	dUTP diphosphatase.
3.6.1.29	Bis(5'-adenosyl)-triphosphatase.
3.6.1.3	Adenosinetriphosphatase.
3.6.1.40	Guanosine-5'-triphosphate,3'-diphosphate diphosphatase.
3.6.1.41	Bis(5'-nucleosyl)-tetraphosphatase (symmetrical).
3.6.1.5	Apyrase.
3.6.1.6	Nucleoside diphosphate phosphatase.
3.6.1.8	ATP diphosphatase.
4.1.1.21	Phosphoribosylaminoimidazole carboxylase.
4.1.1.23	Orotidine-5'-phosphate decarboxylase.
4.3.2.2	Adenylosuccinate lyase.
4.6.1.1	Adenylate cyclase.
5.4.2.2	Phosphoglucomutase (alpha-D-glucose-1,6-bisphosphate-dependent).
5.4.2.7	Phosphopentomutase.
5.4.99.18	5-(carboxyamino)imidazole ribonucleotide mutase.
6.3.2.6	Phosphoribosylaminoimidazolesuccinocarboxamide synthase.
6.3.3.1	Phosphoribosylformylglycinamidine cyclo-ligase.
6.3.4.13	Phosphoribosylamine--glycine ligase.
6.3.4.18	5-(carboxyamino)imidazole ribonucleotide synthase.
6.3.4.2	CTP synthase (glutamine hydrolyzing).
6.3.4.4	Adenylosuccinate synthase.
6.3.5.2	GMP synthase (glutamine-hydrolyzing).
6.3.5.3	Phosphoribosylformylglycinamidine synthase.
6.3.5.5	Carbamoyl-phosphate synthase (glutamine-hydrolyzing).
